# Pupillometric VoE paradigm reveals that 18- but not 10-month-olds spontaneously represent occluded objects (but not empty sets)

**DOI:** 10.1371/journal.pone.0230913

**Published:** 2020-04-24

**Authors:** Wiebke Pätzold, Ulf Liszkowski

**Affiliations:** Department of Developmental Psychology, University of Hamburg, Hamburg, Germany; University of Iowa, UNITED STATES

## Abstract

Object permanence has been investigated with a variety of paradigms and measures, yielding heterogeneous findings. The current study employed a novel Violation-of-Expectation paradigm measuring pupil dilation as indicator of cognitive effort and surprise. Across repeated trials, infants watched videos of animated toys either stopping in an open door frame or moving across the open door frame off screen. The door then closed and opened up again to reveal either the toy, or an empty space. In Experiment 1, 18-month-olds’s pupils dilated in response to the unexpected empty outcome more than to the expected empty outcome, establishing the paradigm as a suitable measure of violation of object expectation. Using the same paradigm, Experiment 2 revealed an absence of this object expectation effect for 10-month-olds. Results are discussed with regard to paradigmatic aspects and developmental differences. It is suggested that young infants do not automatically represent occluded objects upon perceiving occlusion events, and that occlusion events may initially require relevance in terms of individual activity or social interaction.

## Introduction

Object permanence, the ability to represent an object’s continued existence when it is not visible [1] has been the focus of cognitive development research for many decades. Classic behavioral studies reliably found that infants begin to grasp for occluded objects around 8 to 10 months but still commit search errors when the object is visibly transposed to a new hiding location [2–4]. These findings have been criticized, because the dependent measure relies on additional complexities of coordinating action and knowledge and may thus underestimate infants’ competence [5, 6; but see 7]. Subsequent research has used Violation-of-Expectation (VoE) paradigms with looking time as dependent measure, suggesting that object permanence emerges as early as 3 months [8, 9]. However, looking-time VoE studies have been criticized to overestimate infants’ competence because looking-time paradigms may be susceptible to lower level perceptual accounts, e.g. perceptual salience and novelty of the test event [10–12; see 13]. Further, looking time effects may depend on a fragile balance between familiarity vs. novelty preference when varying the number of familiarization trials [14–16]. Dunn and Bremner [17] recently found that six-months-old infants looked longer at test trials with a violation of object expectation, but also at test trials with a novel object. It appears notoriously difficult to distinguish whether an effect of longer looking is driven by violations of object expectations or simply by processing a novel object, one problem being that incorrect outcomes are often inherently novel.

For example, regarding the classic ‘drawbridge’ VoE looking-time paradigm [9] it has been argued that infants may look longer at the impossible test event in which an occluder flips backwards in an 180° arc despite an object standing in its way, because of a preference for some general inherent perceptual properties of the stimuli rather than an object expectations [18, 19]. To test this possibility, Sirois and Jackson [19] used a full factorial design and habituated infants not only to the 180° arc of the moving occluder as in the original study [9], but also to the 120° moving occluder displayed during test events. They found the original looking time effect, i.e. longer looking to the event when the occluder moved 180° backwards despite a box being behind it; however, they also found that overall, infants looked longer at events involving the 180° angle (irrespective of habituation); and at events involving the box (irrespective of the impossibility of the event). Sirois and Jackson therefore concluded that the original looking time effect does not warrant a conclusive interpretation in terms of object permanence.

One way to address these different interpretations of object permanence findings is to employ novel approaches and measures that have the potential to concur on the same interpretation and can remedy paradigmatic challenges. VoE looking-time studies, for example require a prior activation of processing, often to different arbitrary degrees (habituation or familiarization), and the possible and impossible test events often alternate, possibly influencing each other, which makes the investigation of automatic processes less ideal. It is also notoriously difficult to relate looking times of 10 to 30 seconds to real-time cognitive processes and structures. Neurophysiological studies may be especially promising in this regard. For example, two studies measuring oscillatory gamma rhythm in the EEG as a signature for object maintenance during occlusion found positive evidence at 6 months of age [20, 21]. Gamma oscillations typically increase immediately in response to occlusion of objects and the increase is short lived [22]. One interpretation is thus that infants only briefly maintain a trace of the object at the moment it becomes occluded (< 1 sec.), facilitating immediate perception of its reappearance. It is less clear whether this measure also reveals a longer lasting cognitive representation of a stationary object that is occluded for several seconds (e.g., to then guide action). Another promising measure that circumvents the interpretative problems of behavioral reaching and looking time measures is pupil dilation. Because pupils dilate not only to changes in luminance but also with regard to cognitive effort, one can show participants the exact same perceptual event under different cognitive expectations and measure involuntary changes in pupil diameter as a function of cognitive processing, while excluding lower level perceptual effects [23–25]. Research with infants has shown pupil dilation effects as early as 4–8 months in response to pupillary contagion [26], others’ emotional distress [27], detection of phonetic regularities [28], action goal violations [29], and surprising physical events [30], among others, which reveals pupil dilation as a cognitive measure beyond luminance changes (for overviews, see [31, 32]).

Sirois and Jackson [19] measured in their drawbridge replication study both looking times and pupil dilation. In accordance with the perceptual interpretation of their looking time results, the authors did not find a selective effect of pupil dilation for the conditions which should violate cognitive expectations. These findings shed doubts on the robustness of VoE effects of object permanence at 10 months of age. However, Sirois and Jackson [19] did not provide positive evidence (e.g., with older children) that their paradigm and measure of pupil size does work, making it difficult to interpret the absence of evidence for 10-months-olds.

In the current study, we followed up on the pupillometric findings to test whether infants show object permanence in a simplified visual violation-of-expectation task. We did not use the moving drawbridge paradigm to avoid additional demands and potential confounds of processing and preferring different movements of the drawbridge. Further, we did not employ familiarization as by Sirois & Jackson [19], but a multi-trial event-related paradigm to compare average condition differences in phasic changes of the pupil diameter. In a fully balanced 2x2 factorial design, in logic alike to the EEG study by Kaufman et al. [20], but with longer occlusion, we showed infants an occluder that opened to reveal either an object or an empty location. The appearance or disappearance of objects was either expected or unexpected depending on whether an object or an empty location had previously been shown to be behind the occluder. This enabled us to compare perceptually identical outcomes that were thus identical in luminance and differed only in their cognitive expectedness.

Our main question concerned the comparison between the expected and unexpected disappearance of an object. In the unexpected (violation) condition, an object that was present before occlusion failed to appear after occlusion. In contrast, in the expected condition, an object that had disappeared before occlusion remained absent after occlusion. Success would require representing the object during its occlusion and expecting it to reappear. The violation of this expectation should yield larger pupils compared to the expected event.

We prevented infants from always expecting an empty location outcome by alternating the outcome of an empty location and an object across trials. Therefore, a secondary possibility for a comparison arose, regarding the expected and unexpected appearance of an object. Thus, in the unexpected (violation) condition, an object that had already disappeared before occlusion now appeared after occlusion. In contrast, in the expected condition, an object that had been present before occlusion remained there after occlusion. Success would require representing and expecting the ‘nothingness’, or empty set. Pupils should dilate when an object was displayed where an empty location was supposed to be. There is less literature on this topic, but it appears that infants are not surprised to see objects unexpectedly appear compared to when objects disappear unexpectedly. In a looking-time study by Wynn and Chiang [33], eight-months-old infants did not look longer at an unexpected appearance event compared to an expected appearance, both in a scene with two toys (one that was expected to be there, and one that should not have been there) and a scene with a single toy (which should not have been there; for similar results, see [20]. While looking time studies on early numeracy have suggested that infants are sensitive to the subtraction of objects from an occluded scene [34], the concept of emptiness, or zero, appears far from trivial and may not be found until much later in childhood [35, 36; see discussion of 33]. We therefore included the unexpected appearance comparison in our analyses only as a secondary question.

To test our main question, whether pupillometry would reveal object permanence in infants, we tested in Study 1 an older age group of 18-month-olds, an age expected to reveal firm object permanence. 18-month-olds’ pupils should be larger in the unexpected condition when an object which should be there had suddenly disappeared, compared to the identical outcome scene when it was expected that the object should not be there. In Study 2, we moved on to our target age group of 10-month-olds for whom findings of object permanence are more heterogeneous.

## Experiment 1

### Methods

This research was approved by the ethics committee of the Institute for Psychology, Hamburg University (approval no.: 2017_141 Paetzold).

#### Participants

Nine-teen 18-months-old infants (10 males, 9 females) were included in the final sample. Mean age was 18 months, 13 days (range: 18 months, 3 days– 18 months, 26 days). Six additional infants participated (3 males, 3 females) but were excluded from the sample due to failure in reaching minimum looking times during manipulation (4), fussiness (1) or technical failure (1). On average, infants provided data for 14 trials of out 16 trials (range 6–16).

All infants were recruited via birth records and had a middle-to-high socioeconomic, western cultural background. Infants were included in the final analysis when they provided data for at least one trial of each condition. Trials were included when looking times indicated that the child had watched at least 50% of the manipulation.

#### Apparatus

Pupil dilation was measured with a Tobii x120 eye tracker (Tobii Technology, Stockholm, Sweden) which was attached to a standard computer screen. The presentation screen and the eye tracker were placed in a testing booth built for this purpose, with black canvases behind and on both sides of the screen. A video camera above the screen allowed the researchers to monitor the infant’s behaviour during the session. The size of the stimuli presentation was 1280 x 1024 px on a 1920 x 1200 px screen, with the rest of the screen appearing black throughout the experiment. The size of the display was 34.50 x 27.50 cm on the screen, which corresponds to a visual angle of 24.25° horizontally and 30.16° vertically.

#### Stimuli

**Fig 1:**
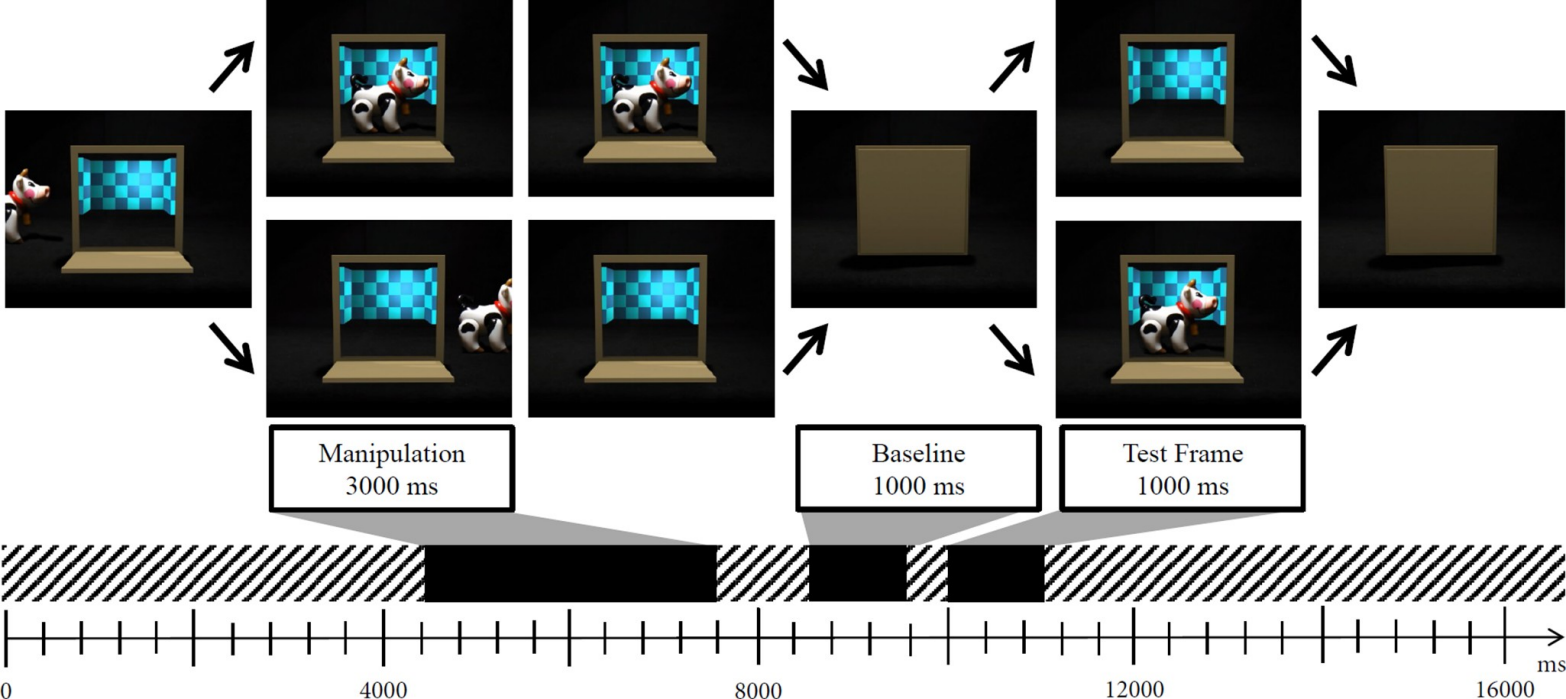
Depiction of animated stimuli and experimental design.

The videos consisted of computer-animated clips of a stage-like scene (see Fig 1). Centered in the video was a closed light-brown door frame. All videos started with the door opening from top to bottom to reveal the empty inside, which was lined with a bright turquoise checking pattern (500 ms).

The pattern implicated depth inside the frame and also served to balance luminance differences between an empty outcome and an outcome containing one of four toys: a boat, a cow, a train and a horse. Immediately following the complete opening of the door, one of the toys started moving into the screen from one side (left/right was counterbalanced so that each child saw each condition from each side the same amount of time). The movement was accompanied by a sound typically associated with that toy in an animation (boat gliding on water for the boat, cow bells for the cow, railroad for the train, clip-clopping of hooves for the horse). After 3750 ms, the toy had moved into the middle of the screen. In half of the videos, the toy remained in the middle of the screen until it was occluded by the closing door (3250 ms). In the other half of the videos, the toy did not stop but continued its trajectory until it was completely out of sight. As it was leaving the screen, the door closed. The timing of the door closing was matched so that in both versions, infants saw the toy the exact same amount of time. In all videos, the second part of the toy animation—staying still or moving out—was accompanied by another toy-specific sound (fog horn for the boat, mooing for the cow, choo-choo for the train, neighing for the horse). The door then remained closed for 2000 ms before opening again (500 ms), accompanied by the sound of a drumroll, to reveal the outcome: either the same toy as before or no toy at all. The outcome was presented for 2000 ms before the door closed again (500 ms) and stayed closed for the remainder of the video (4000 ms). Sound was included to keep infants engaged with the videos. The timing of sounds was identical in all conditions.

Infants were presented with 16 videos, four of each condition. Appearance of toys was blocked in the same order for all infants, whereas order of conditions was semi-random: no same manipulation or outcome could be presented more than twice in a row. Each of the infants saw each toy in all four conditions, at different times in the study. Before each video, a short attention getter was played to redirect the child’s attention towards the middle of the screen (3s). After a block of four videos, a longer attention getter was played to break up the routine of the videos (8s).

Because we averaged data from several trials with different objects, we took care to match luminance levels of the four objects as closely as possible. Following the formula used by Jackson and Sirois [30], we calculated photometric luminance from the red, green and blue levels of the images as given by a photo analysis software. On a scale from 0 to 1 with 1 being the brightest, luminance levels read .252 for the empty box, .272 for the boat, .261 for the cow, .236 for the train and .241 for the horse. Thus, the biggest difference in luminance was between the train and the boat with the boat being 3.7% brighter than the train. The empty box was exactly at the mean of luminance of the full boxes.

#### Procedure

The experimenter explained the study to the caregiver and obtained their informed consent. The caregiver was seated on a swivel chair in the testing booth 64 cm away from the presentation screen with the child on his or her lap. A 9-point-calibration ensured that the child’s eyes were properly captured by the eye tracker. The experimental stimuli were then presented on the screen as long as the child was willing to watch or until all 16 trials were completed. The caregivers were instructed to neither talk to nor point for the child and to keep their eyes closed for the duration of the experiment.

#### Data processing

Pupil size and gaze location of both eyes were recorded at a 120 Hz rate. Pupil size from the left and right eye was averaged to mean pupil size. If data from one eye was missing, the data from the other eye was used. If data from both eyes were missing, no substitution was made.

Gaze data was processed in relation to an a priori defined area of interest (AOI) of the door frame (500 x 500 px). Gaze data at the entire display (1280 x 1024 px) was also calculated in order to allow analysis of looking time. Time looked away from the screen was defined as (Maximum Looking Time—Looking Time to Display).

After careful visual inspection, time windows for analyses of pupil size were set to 500ms after the event boundaries of the stimuli. Infants’ pupils react slower than adults’ [37] and therefore need more time to adapt to a change in stimulus. Also, by not including the phases of pupillary constriction (PC) following a stimulus onset in the average, a more accurate representation of the tonic pupil dilation is achieved [32].

Because pupil data as collected by remote eye trackers such as the Tobii X120 may be susceptible to error introduced by gaze direction [38], we checked that the gaze patterns did not differ systematically from one condition to the other during the test phase. 68.89% of all gaze points (range = 64.40%-73.55%) fell into the central AOI of the door area during the test event, with no significant differences between conditions (*F*(1,18) = 2.649, *p* = .084, *η*_*p*_^2^ = .332).

### Results

#### Preliminary analyses

**Fig 2:**
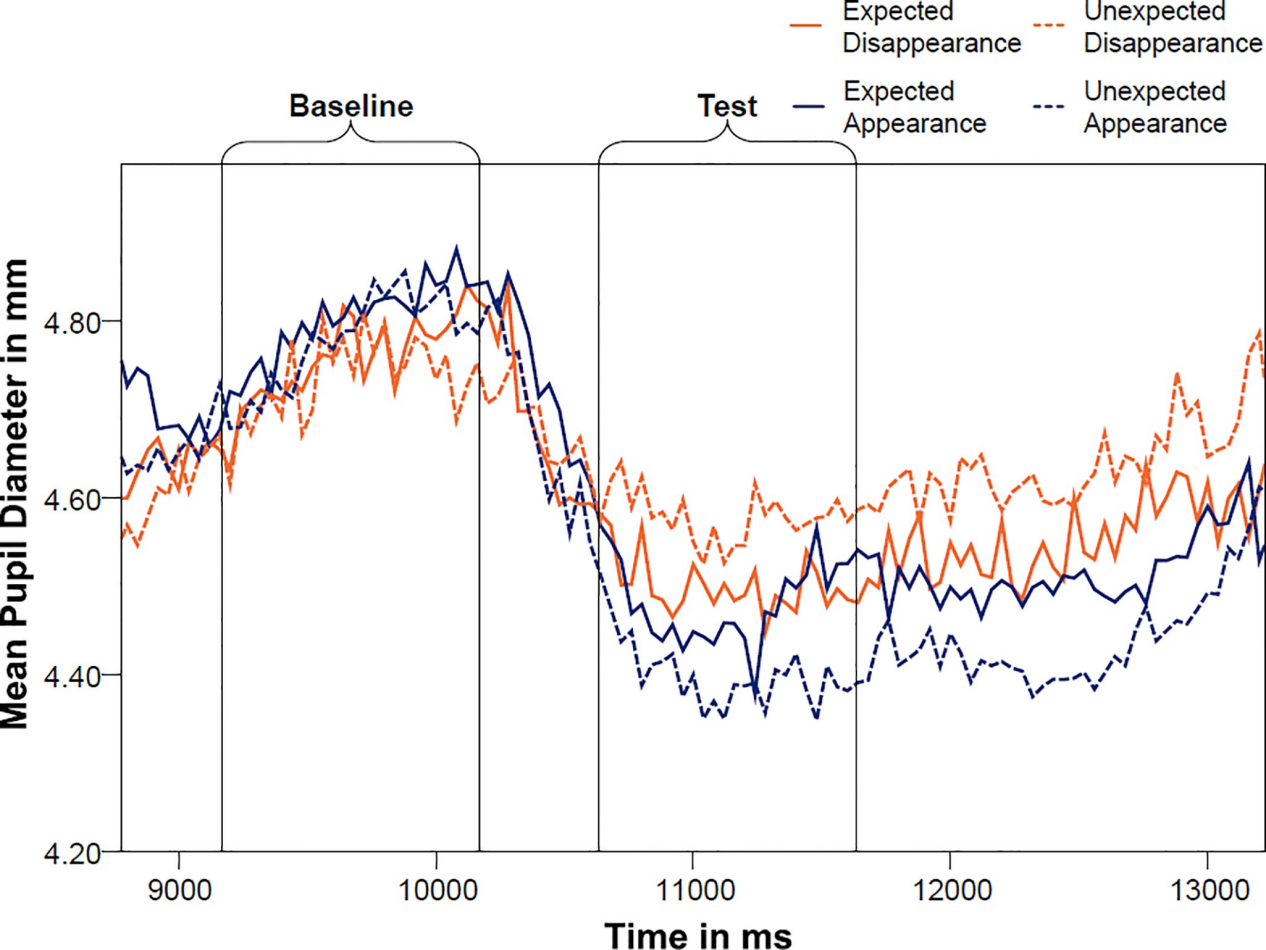
Mean pupil diameter of 18-months-olds across baseline and test for the four conditions. Time windows are corrected for the time lag in pupil change (+500ms).

**Fig 3:**
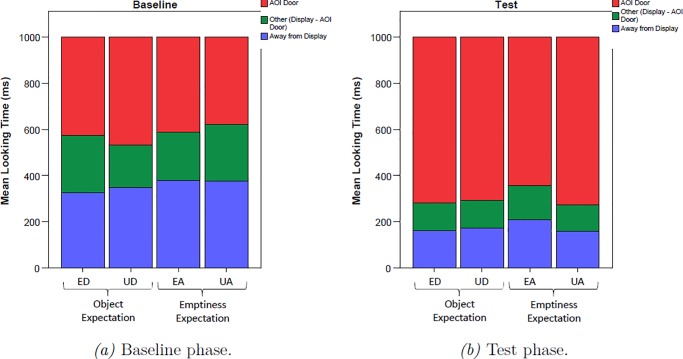
Looking times of 18-months-olds to the door occluder; to the entire display; and away from the display (i.e. looks not registered on the display) during 1000ms baseline (a) and first 1000ms test (b).

Fig 2 displays the time line of pupil dilation during baseline and test. A priori analyses of the baseline revealed that there was no difference in pupil dilation between the two types of manipulation at baseline (M_occlusion_ = 4.83mm, SD_occlusion_ = .546 mm, M_removal_ = 4.87mm, SD_removal_ = .527mm, *t*_(28)_ = .832, *p* = .416). Fig 3a further shows that infants looked during baseline for the same amount of time at the video, with no significant differences between the two types of manipulation (M_occlusion_ = 622.49 ms, SD_occlusion_ = 206.30 ms, M_removal_ = 640.37 ms, SD_removal_ = 177.99 ms, *t*_(28)_ = .570, *p* = .576).

#### Main analyses

**Fig 4:**
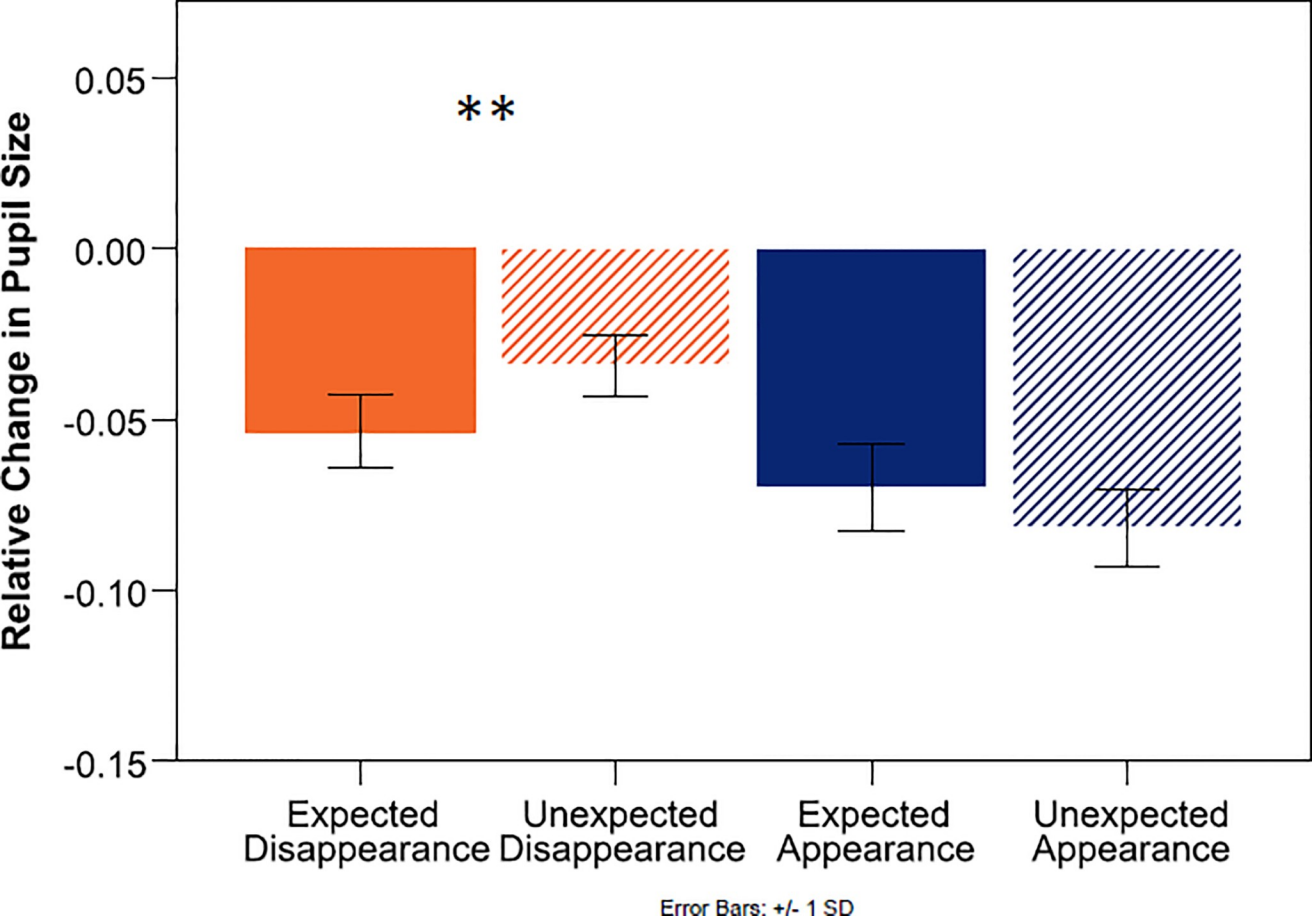
Mean change in pupil size relative to baseline in 18-months-olds. Note that a smaller negative deviation from zero denotes a larger pupil.

Fig 4 shows the relative change of pupil size at test, averaged over the duration of the first 1000 ms. A 2x2 (Outcome x Expectation) repeated-measures ANOVA revealed a main effect for Outcome (*F*_(1, 18)_ = 19,39, *p*< .000, *η*_*p*_^2^ = .519), no main effect for expectation, and a significant interaction between outcome and expectation (*F*_(1, 18)_ = 12.97, *p* = .002, *η*_*p*_^2^ = .419). Regarding our hypothesis about the disappearance of the object, direct comparisons showed that infants’ pupils dilated to the empty outcome significantly more when it was unexpected than when it was expected (expected disappearance = ED, unexpected disappearance = UD. M_ED_ = -.0535, SD_ED_ = .04614, M_UD_ = -.0341, SD_UD_ = .03909, *t*_(18)_ = 3.239, *p* = .005). Regarding the secondary question about the appearance of the object, there was no difference in infants’ pupil sizes in response to the toy outcomes (expected appearance = EA, unexpected appearance = UA, M_EA_ = -.0700, SD_EA_ = .05636, M_UA_ = -.0817, SD_UA_ = .04848, *t*_(18)_ = 1.547, *p* = .139).

#### Control analyses

To make sure that differences in pupil size did not arise from different perceptual stimulation, we analyzed whether infants did look to the door AoI for the same amount of time during the test phase (Fig 3b). Infants looked at the videos for the same amount of time during the first 1000 ms of the expected and unexpected empty outcomes (M_ED_ = 812.06 ms, SD_ED_ = 237.94 ms, M_UD_ = 790.20 ms, SD_UD_ = 224.44 ms, *t*_(18)_ = 2.059, *p* = .304) and during the expected and unexpected toy outcomes (M_EA_ = 785.16 ms, SD_EA_ = 238.91 ms, M_UA_ = 835.31 ms, SD_UA_ = 212.65 ms, *t*_(18)_ = .953, *p* = .353). There were also no differences in looks to the door or looks away from the screen when comparing the expected and unexpected outcomes (p > .127).

### Discussion

The current results reveal that infants’ pupils increase in response to an unexpected disappearance of an object, i.e. when an object does not re-appear after a temporary occlusion. Importantly, infants’ pupils dilated significantly less when the object expectedly did not appear after occlusion (because it had already disappeared before occlusion). This excludes the possibility that purely perceptual differences drove the effect. Our further control analyses confirmed that the effect was neither due to differences in pupil sizes at baseline nor explainable by differences in visual attention during baseline or test. Further, the two second delay from occlusion to outcome excludes an interpretation in terms of an immediate short-lived perceptual effect. The pattern of findings supports an interpretation in terms of object permanence and provides confirmatory evidence to the established view of object representations in the second year of life. The new paradigm thus proves to be a useful test in detecting object permanence while it circumvents the interpretative challenges of habituation paradigms and looking time measures.

The current experiment did not find a differential effect of pupil dilation to the unexpected appearance of an object, i.e. when an object appeared after temporary occlusion of an empty location. Given that our control measures did not reveal any differences that could have accounted for the absence of the effect, we assume that 18-month-olds do not represent empty sets, at least in the current paradigm. While the absence of evidence certainly does not reveal evidence for absence, the lack of an effect for the unexpected appearance has to be interpreted in the context of our positive effect for the unexpected disappearance. In line with the current literature [39; see also 20, 33], we therefore think that the representation of empty sets is beyond 18-month-olds’ capacities, at least in spontaneous settings, and when compared to their ability to represent occluded objects, as our result of an interaction must suggest.

## Experiment 2

Given that our paradigm revealed object expectations at 18 months, in Experiment 2 we moved on to test the more contested target age group of 10-month-olds, to assess whether they, as a group, would show evidence of automatic object expectations in our new paradigm, and how it would compare to the current results with the 18-month-old infants. If 10-month-olds indeed represent occluded objects in our paradigm, their pupils should dilate significantly more in the unexpected disappearance event compared to the expected disappearance event. Because the effect could be smaller in younger infants we increased our sample size. As in Experiment 1, in order to prevent infants from always expecting an empty outcome we included again the toy outcome in a full-factorial 2x2 design. However, given that the 18-month-olds did not show evidence of representing the empty set in Experiment 1, we did not expect that 10-month-olds would represent the empty set in the current experiment.

### Methods

#### Participants

Twenty-eight 10-months-old infants (14 males, 14 females) were included in the final sample. Mean age was 10 months, 13 days (range: 10 months, 2 days– 10 months, 30 days). Four additional infants participated (2 males, 2 female) but were excluded from the sample due to failure in reaching minimum looking times during manipulation (1), pupil size more than two standard deviations larger than mean (1) or technical failure (2). On average, infants provided data for 13 trials of out 16 trials (range 5–16).

Recruitment and inclusion criteria were identical to Experiment 1. Apparatus, Stimuli, Procedure and Data Processing were identical to Experiment 1. On average, 73,69% of all gaze points (range = 70,47%-76,22%) fell into the central AOI of the door area during the test event, with no significant differences between conditions (F(1,27) = 1.337, p = .285, *η*_*p*_^2^ = .138).

### Results & discussion

#### Preliminary analyses

**Fig 5:**
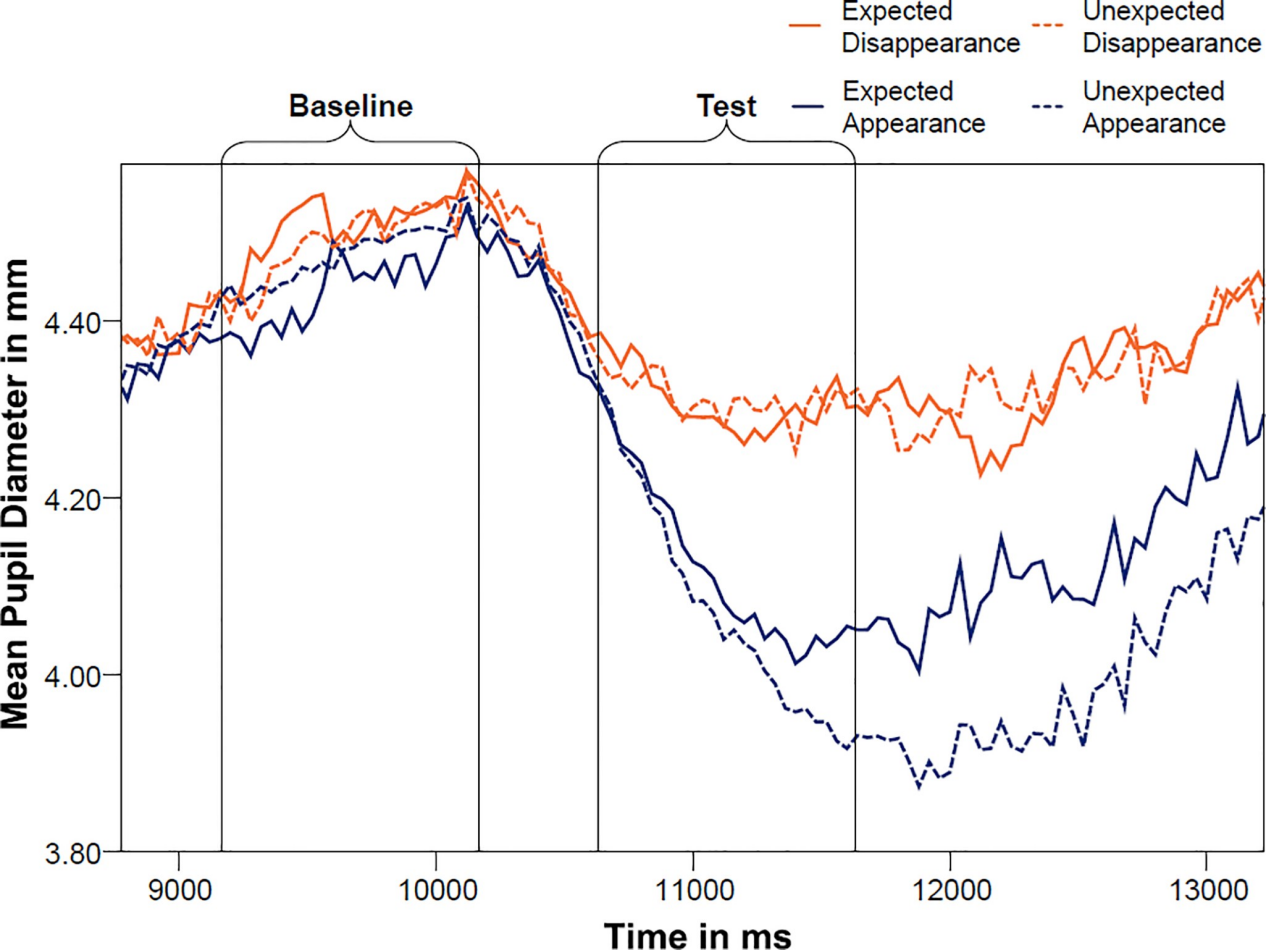
Mean pupil diameter of 10-months-olds across baseline and test for the four conditions. Time windows are corrected for the time lag in pupil change (+500ms).

**Fig 6:**
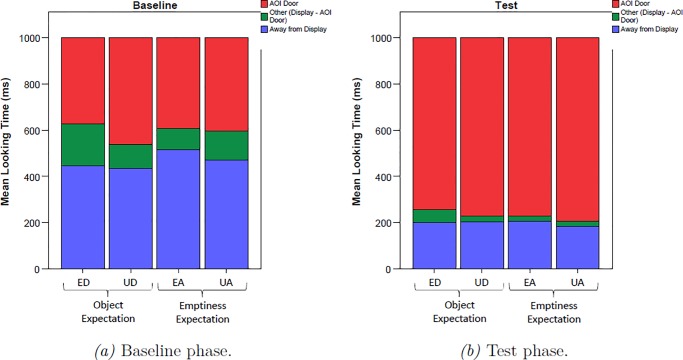
Looking times of 10-months-olds to the door occluder; to the entire display; and away from the display (i.e. looks not registered on the display) during 1000ms baseline (a) and first 1000ms test (b).

Fig 5 displays the mean pupil diameters for the four conditions during baseline and test. Visual inspection of the time line of pupil dilation revealed no apparent differences between conditions during the baseline phase. Paired *t*-tests confirmed that pupil dilation was not different during the two types of manipulation (M_occlusion_ = 4.55 mm, SD_occlusion_ = .540 mm, M_removal_ = 4.56mm, SD_removal_ = .507 mm, *t*_(27)_ = .555, *p* = .584). Fig 6a further reveals that infants looked at the screen the same amount of time, irrespective of which manipulation they had previously seen (M_occlusion_ = 527.10 ms, SD_occlusion_ = 173.32 ms, M_removal_ = 543.98 ms, SD_removal_ = 195.20 ms, *t*_(27)_ = .648, *p* = .522). Thus, infants were paying the same amount of attention towards the screen during baseline, with no significant difference in pupil size, assuring that the calculation of the relative change of pupil size to the test outcomes was unbiased by differences in the baseline.

#### Main analyses

**Fig 7:**
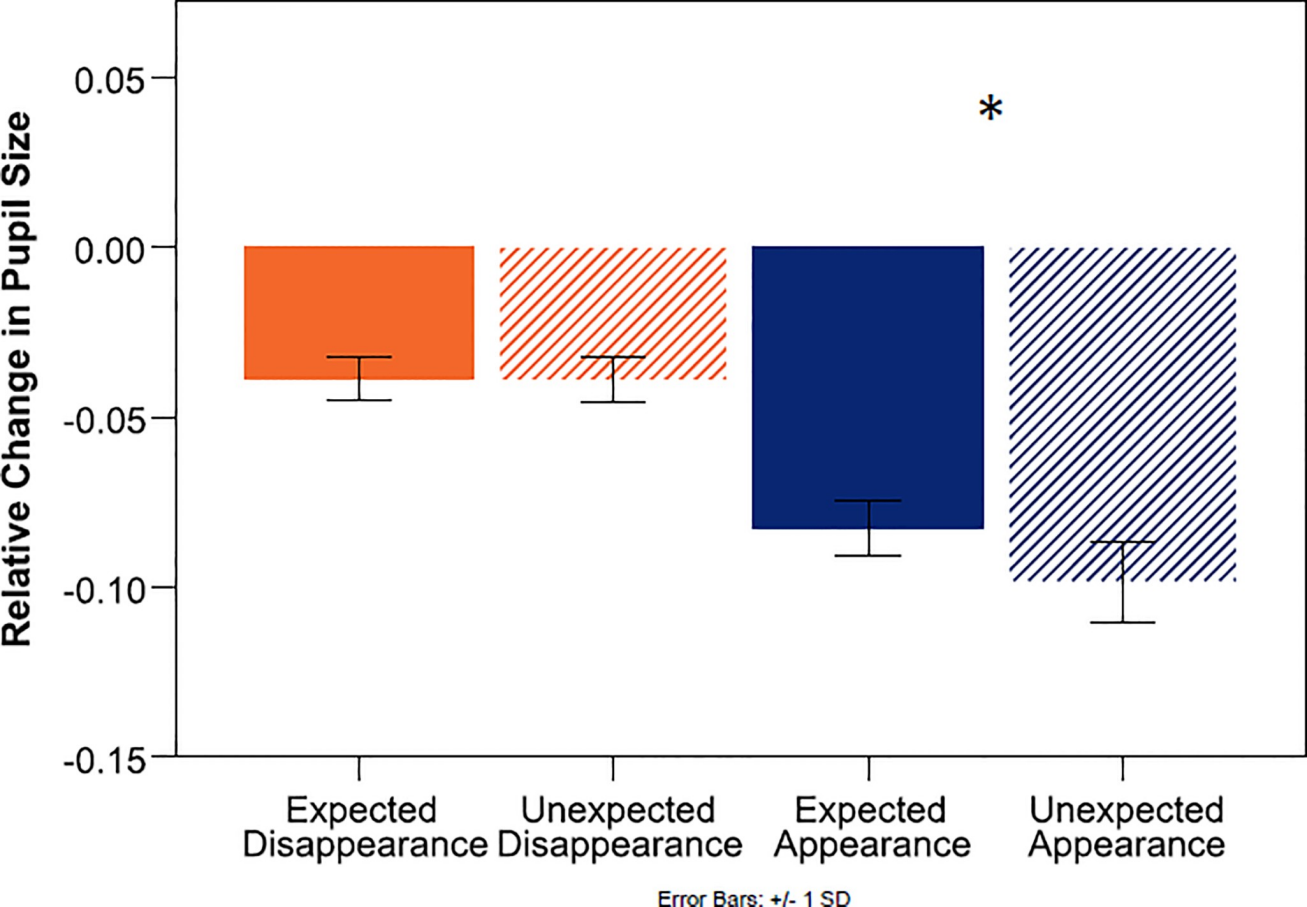
Mean change in pupil size relative to baseline in 10-months-olds. Note that a smaller negative deviation from zero denotes a larger pupil.

Fig 7 shows the results of the relative change score analysis at test, averaged over the duration of the first 1000 ms. A 2(outcome) x 2(expectation) repeated-measures ANOVA revealed a main effect for Outcome (*F*_(1, 27)_ = 59.31, *p*< .000, *η*_*p*_^2^ = .687), such that infants’ pupils dilated more to the empty outcome compared to the toy outcome, and no main effect for expectation. Unlike for the 18-month-olds, the interaction term did not reach significance (*F*_(1, 27)_ = 1.92, *p* = .177, *η*_*p*_^2^ = .067).

The direct comparison following our hypothesis further confirmed that infants’ pupils indeed did not dilate differently between the expected and unexpected empty outcomes (M_ED_ = -.0385, SD_ED_ = .03322, M_UD_ = -.0390, SD_UD_ = .03644, *t*_(27)_ = .064, *p* = .475, 1-tailed). Although we did not expect differences between the toy outcomes, and the interaction term did not reach significance, we also report the comparison between the expected and unexpected object appearance, for completeness. Surprisingly, the expected appearance event yielded a relatively larger increase in pupil size than the unexpected appearance event (M_EA_ = -.0825, SD_EA_ = .04274, M_UA_ = -.0986, SD_UA_ = .06235, *t*_(27)_ = 2.182, *p* = .038). While there is currently no clear interpretation of this side effect and its unexpected reverse direction, we must note that in the absence of a significant interaction term and any hypothesis predicting this direction, the finding does not remain significant after an alpha-level correction, which would be appropriate for the post hoc comparison, and might thus be spurious.

#### Control analyses

Fig 6b displays results of our control analyses of the looking times during the test phase. Infants looked at the videos for the same amount of time during expected and unexpected empty outcomes (M_ED_ = 704.66 ms, SD_ED_ = 310.20 ms, M_UD_ = 736.66 ms, SD_UD_ = 266.01 ms, *t*_(27)_ = .679, *p* = .503) and during expected and unexpected toy outcomes (M_EA_ = 743.95 ms, SD_EA_ = 279.53 ms, M_UA_ = 762.15 ms, SD_UA_ = 259.06 ms, *t*_(27)_ = .758, *p* = .455). Similarly, there were no differences with regard to looks towards the door and looks away from the screen when comparing the expected and unexpected outcomes (p > .455). These results thus reveal no additional factor in our data that could account for the pupillometric findings. Notably, our measurement was sensitive enough to detect significant differences in infants’ pupil sizes, as revealed by the main effect for the outcome, excluding the possibility that the pupillometric measure did not work at all.

Current results thus suggest that 10-month-olds do not form object expectations following occlusion, which is in line with the pupillometric and looking time findings by Sirois and Jackson [19]. The current design did not use familiarization or habituation, and it presented multiple trials with immediate, single condition test events, unlike VoE looking time paradigms, which makes it a stronger case for the current design. The absence of the effect in our current paradigm is especially meaningful given the presence of the effect at 18 months of age. For sake of completeness, when comparing object expectation between experiments in a 2 (expected vs. unexpected disappearance) by 2 (Experiment 1 vs. 2) ANOVA revealed no main effect of Experiment (F(1,46) = .253, *p* = .617, *η*_*p*_^2^ = .006), a reduced effect of object expectation relative to that at 18 months (F(1,46) = 3.1, *p* = .086, *η*_*p*_^2^ = .064), and an interaction with Experiment in the predicted direction (F(1,46) = 3.438, *p* = .070, *η*_*p*_^2^ = .071), supporting the a priori approach of our planned experimental comparisons for each age group separately. Analyses of the two experiments then shows that the effect of object expectations was driven entirely by the 18-month-olds, and absent at 10 months of age.

## General discussion

The current study employed a new Violation-of-Expectation paradigm using pupil size as a measure of object representation during occlusion to add to a field of heterogeneous findings on object permanence in the first year of life. The paradigm revealed spontaneous object expectations in 18-month-olds, proving that the paradigm is sensitive to assessing violations of object expectations in infants. Regarding the more contested age of 10 months, however, the paradigm revealed no evidence for spontaneous expectations of objects during occlusion. What can we make of 10-month-olds’ failure in light of 18-month-olds’ passing in the current object permanence paradigm?

On the one hand, our findings of 10-month-olds’ failure is in line with previous findings. Most notably, our findings concur with the pupillary findings by Sirois and Jackson [19] and provide confirmatory support for the absence of a pupil dilation effect at 10 months of age. The current study substantiates this absence of evidence by presenting positive evidence with the same paradigm for an older age group. In combination with leaner interpretations of looking time VoE studies in terms of perceptual preferences and biases rather than cognitive representations, current findings add to doubts about automatic representations of occluded objects early in the first year of life.

Other VoE looking time paradigms similar in logic to the current one [20, 33] have produced positive findings in younger infants. On a paradigmatic level, one difference is that in looking time studies, the different condition test-events alternate sequentially, one after the other, possibly influencing each other in terms of order, and increasing salience of the condition contrasts. Further, the time of occlusion in the Kaufman et al. [20] study was rather short (in the visual VoE experiment about 1 second; in the EEG experiments about 500ms). Finally, familiarization provides relevant prior information against which violations are then assessed, which is not the case in multiple trial designs that rather measure automatic processing.

On the other hand, the lack of evidence at 10 months appears to be at odds with action-based research. Modified reaching studies reveal that 6-month-olds can reach for objects in the dark [40, 41] or in opaque liquids [42], suggesting that their immediate reaching (without, or little, delay) is guided by representations of invisible objects. However, their reaching in the dark appears still different from that of adults [43], and reaching for occluded entities remains less proficient. While it has been suggested that occlusion events may be especially hard, even for adults [13], an anticipatory looking study [44] found that 4-month-olds anticipate a communicating agent to retrieve an object by looking to one of two occluders behind which they had last seen the object (after a 2-second-delay, not after an 8-second-delay).

On a paradigmatic level, a difference between the aforementioned action-based and anticipatory looking paradigms and the current paradigm is that these did not involve violations of expectations. Further, in the action-based paradigms employing reaching and anticipatory looking, infants are first prepared to execute these behaviors in familiarization trials, to ensure that infants will understand the scenario with regard to acting in it. The test scenario thus gains prior relevance from acting in it. In the current pupillometric VoE paradigm there was no such familiarization since the neurophysiological measure does not require acting, and automatic processes should be less susceptible to prior relevance. One possibility why 10-month-olds’ did not show the effect in the current paradigm is then that they simply did not process the relevance of the scene in terms of the object’s permanence and its violation. This proposal entails that task-inherent demands masked an existing competence in 10-month-olds. Any explanation for 10-month-olds’ failure must of course be seen in light of 18-month-olds’ competent performance in the task and should thus invoke developmental change in infants’ stability of representing of occluded objects.

On a conceptual level, a possible interpretation is then that young infants do not automatically represent occluded objects. Representing occluded objects may initially require more relevance. One source of relevance may be to prepare for action, for example when grasping a moving object along an opaque trajectory, as simplified reaching studies suggest [45]. From an ecological perspective, young infants may only occasionally search for objects (e.g. a lost pacifier), but by 18 months it will be of greater relevance to keep track of objects for a variety of reasons. In contrast to action accounts, other accounts propose that visual experience is sufficient to instigate representations of occluded objects [46], but it is less clear on these views under which circumstances visual experiences of occlusion gain their relevance. Another source of relevance, we suggest, is social in nature, for example when infants’ attention is directed to locations of occluded objects [47]. In a baby’s life it is easy to see how social situations abound. Caregivers’ spent a great deal of activity showing objects to their infants. It remains to be tested what the relative contributions of individual search experience [see 48] and social interactions are for the relevance of spontaneously forming cognitive representations of occluded objects.
